# High Interstitial Fluid Pressure Is Associated with Low Tumour Penetration of Diagnostic Monoclonal Antibodies Applied for Molecular Imaging Purposes

**DOI:** 10.1371/journal.pone.0036258

**Published:** 2012-05-08

**Authors:** Markus Heine, Barbara Freund, Peter Nielsen, Caroline Jung, Rudolph Reimer, Heinrich Hohenberg, Uwe Zangemeister-Wittke, Hans-Juergen Wester, Georg H. Lüers, Udo Schumacher

**Affiliations:** 1 Institute of Anatomy and Experimental Morphology, University Medical Center Hamburg-Eppendorf, Hamburg, Germany; 2 Department of Biochemistry and Molecular Cell Biology, University Medical Center Hamburg-Eppendorf, Hamburg, Germany; 3 Department of Diagnostic and Interventional Radiology, University Medical Center Hamburg-Eppendorf, Hamburg, Germany; 4 Heinrich-Pette-Institute for Experimental Virology and Immunology at the University of Hamburg, Hamburg, Germany; 5 Institute of Pharmacology, University of Bern, Bern, Switzerland; 6 Pharmaceutical Radiochemistry, Technical University Munich, Garching, Germany; National Cancer Institute, NIH, United States of America

## Abstract

The human epithelial cell adhesion molecule (EpCAM) is highly expressed in a variety of clinical tumour entities. Although an antibody against EpCAM has successfully been used as an adjuvant therapy in colon cancer, this therapy has never gained wide-spread use. We have therefore investigated the possibilities and limitations for EpCAM as possible molecular imaging target using a panel of preclinical cancer models. Twelve human cancer cell lines representing six tumour entities were tested for their EpCAM expression by qPCR, flow cytometry analysis and immunocytochemistry. In addition, EpCAM expression was analyzed *in vivo* in xenograft models for tumours derived from these cells. Except for melanoma, all cell lines expressed EpCAM mRNA and protein when grown *in vitro*. Although they exhibited different mRNA levels, all cell lines showed similar EpCAM protein levels upon detection with monoclonal antibodies. When grown *in vivo*, the EpCAM expression was unaffected compared to *in vitro* except for the pancreatic carcinoma cell line 5072 which lost its EpCAM expression *in vivo*. Intravenously applied radio-labelled anti EpCAM MOC31 antibody was enriched in HT29 primary tumour xenografts indicating that EpCAM binding sites are accessible *in vivo*. However, bound antibody could only be immunohistochemically detected in the vicinity of perfused blood vessels. Investigation of the fine structure of the HT29 tumour blood vessels showed that they were immature and prone for higher fluid flux into the interstitial space. Consistent with this hypothesis, a higher interstitial fluid pressure of about 12 mbar was measured in the HT29 primary tumour via “wick-in-needle” technique which could explain the limited diffusion of the antibody into the tumour observed by immunohistochemistry.

## Introduction

Detection of cancer cells and more specifically the characterisation of metastatic cancer cells in a non-invasive way is a primary aim of molecular imaging. Several approaches such as Magnetic Resonance Imaging (MRI), Positron Emission Tomography (PET), and Optical Imaging techniques were invented for this purpose. All applications require specific probes against antigens that are highly expressed on the surface of human cancer cells. For promising probes, a specific tumour uptake *in vivo* has been demonstrated after systemic administration via intravenous application in appropriate animal models [1]. The overall probe biodistribution and more specifically the histological distribution of the bound probe within the tested tumour should be analysed in these models.

The epithelial cell adhesion molecule (EpCAM; CD326) is membranous 38-kDa glycoprotein which is highly expressed in cancer tissue of different entities and to a lower extent by normal epithelium [2], [3]. Elevated EpCAM expression was confirmed amongst other tumour entities in breast, pancreatic, colon, prostate and lung cancer [4], [5], [6], [7]. The impact of high EpCAM expression on patient’s survival is still an ongoing debate. High EpCAM expression was associated with poor survival rates for breast, gall bladder and squamous cell carcinoma of the esophagus whereas better survival rates were reported for renal cell carcinoma and pancreatic cancer [8], [9], [10], [11], [12]. The correlation of EpCAM expression and clinical outcome therefore depends on the cancer entity.

EpCAM was the first target for monoclonal antibody therapy against human cancer. Furthermore, the first successful antibody based therapy judged by of overall survival was achieved using an anti EpCAM antibody [13], [14]. Several studies for non-invasive monitoring of cancer cells in xenograft mouse models with EpCAM as target were published over the last 5 years. The metastatic behaviour of human pancreatic cancer cells to lymph nodes were investigated using a near-infrared fluorophore labelled EpCAM [15]. A study with a mouse xenograft model showed that fluorescent intravital live microscopy with a probe against EpCAM antigen could successfully be used for monitoring tumour resection *in vivo* [16]. HNSSC (Squamous cell carcinoma of the head and neck) xenotransplanted severe combined immunodeficient (SCID) mice were treated with radioimmunotherapy against EpCAM positive cells and biodistribution of EpCAM-directed monoclonal antibodies were investigated in EpCAM-transgenic mouse models [17], [18].

To investigate the capabilities and limitations of molecular imaging based on specific antibodies we developed murine xenograft models for *in vivo* detection of EpCAM using the monoclonal antibody MOC31.

This contribution describes the expression of EpCAM in 12 human cancer cell lines *in vitro* and *in vivo* in related primary tumours that were developed in xenograft models. With one of these models we also investigated the accessibility of EpCAM to antibodies in the primary tumour after i.v. application of the anti EpCAM antibody MOC31. We have analyzed the distribution of the MOC31 antibody as well as the interstitial fluid pressure (IFP) in these tumours since enhanced IFP represents an obstacle for efficient delivery of i.v. applicated drugs [19], [20]. Our results indicate that EpCAM expression is wide-spread over all tumours used making it an ideal target for imaging/therapeutic purposes. However, if MOC31 is applied i. v., binding of MOC31 was limited to tumour cells around blood vessels. The increased IFP in tumours could explain the limited distribution over the entire tumour volume. Lowering IFP could therefore be essential to increase the tumour penetration of i. v. applied antibodies directed against tumour antigens.

## Materials and Methods

### Cell Lines

The human prostate cancer cell lines LNCAP and PC3 (both established from metastatic adenocarcinomas) were obtained from the German Collection of Microorganisms and Cell Culture (DSMZ, Germany). The human breast cancer cell lines T47D and MCF7 (both established from pleural effusions) were obtained from European Cell Culture Collection (Porton Down, Wiltshire, UK). The human melanoma cell lines MEWO [21] and FemX-1 [22] (both established from metastatic melanoma lymph nodes) were kindly provided by the Klinik für Dermatologie, Universitätsklinikum Hamburg-Eppendorf, Germany. The human colon cancer cell line HT29 (established from a primary carcinoma of the colon) was obtained from Cell Lines Service (Germany). The human colon cancer cell lines Caco2 and SW480 (both established from a primary adenocarcinoma of the colon) were obtained from European Cell Culture Collection (Porton Down, Wiltshire, UK). The human small cell lung cancer cell line OH-1 (established from pleural effusion) was kindly provided by Prof. Uwe Zangemeister-Wittke, University of Bern, Department of Pharmacology [23]. Two human pancreatic cancer cell line 5061, established from an advanced pancreatic adenocarcinoma and 5072 m, established from an advanced pancreatic adenocarcinoma from a 71-year-old Caucasian woman, were kindly provided by the Klinik und Poliklinik für Allgemein-, Viszeral- und Thoraxchirurgie, Universitätsklinikum Hamburg-Eppendorf, Germany [24]. Written informed consent of the patient for the removal of tissue samples for investigational purposes was obtained prior to surgery. The study was approved by the ethical committee of the Medical Council of Hamburg (Ärztekammer), Germany.

The LNCAP, PC3, T47D, MCF7, MEWO, FemX-1, HT29, Caco2, SW480, OH-1 cells were cultured *in vitro* under standard cell culture conditions (37°C, 100% relative humidity, 5% CO2) in RPMI medium (Gibco/Life Technologies, Paisley, Scotland) supplemented with 10% heat inactivated fetal bovine serum (FBS, Gibco), 2 mM L-glutamine (Gibco), 100 U/ml penicillin and 100 µg/ml streptomycin (Gibco). The cells 5061 and 5072 were cultured in complete (TUM) RPMI 1640 medium with Glutamax (Invitrogen, NY, USA) supplemented with 10% of fetal calf serum (FCS), 200 IU/ml of penicillin-streptomycin, 0.1 mg/ml gentamycin (Biochrom AG, Berlin, Germany), 50 mM of human transferrin (Sigma-Aldrich, Steinheim, Germany), 0.01 µg/ml of bovine insulin (Sigma-Aldrich, Steinheim, Germany), 0.01 µg/ml of recombinant human epidermal growth factor (Pepro Tech, London, UK), and 0.01 µg/ml of human basic fibroblast growth factor (Pepro Tech, London, UK). Before reaching confluence, cells were routinely harvested for passaging using 0.05% trypsin-0.02% EDTA (Gibco).

### Real-time PCR

To quantify EpCAM mRNA amount in relation to glyceraldehyde 3-phosphate dehydrogenase (GAPDH) mRNA amount of the human tumour cells, real-time PCR was performed. In brief, total RNA from tumour cells was isolated using RNeasy Mini Kit (Qiagen, Hilden, Germany) according to the manufacturer’s instructions. The RNA was eluted in 50 µl RNase free water. The RNA-concentration was measured and the quality was checked on a NanoDrop® ND-1000 Spectrophotometer (Peqlab, Erlangen, Germany). The cDNA synthesis was performed in a Biometra thermal cycler (Biometra, Göttingen, Germany) in a total volume of 20 µl for each sample and followed the manufacturer’s instruction for the First Strand Transcriptor cDNA Synthesis Kit (Roche Diagnostics, Mannheim, Germany). Two parallel cDNA approaches were used for reverse transcription separately, with anchored-oligo(dT)18 and random hexamer primer. Two µg of total RNA were used for each cDNA approach and were pooled afterwards. The oligo-dT primers transcribe the mRNA with poly-A sequences and the random primed cDNAs transcribe fragmented RNA. The pooled primers cover therefore all putative EpCAM transcripts. Real-time polymerase chain reaction was performed in a 96 well format with the LightCycler® 480 System (Roche Diagnostics GmbH, Mannheim, Germany). For the real-time PCR, the LightCycler Fast Start DNA MasterPLUS SYBRGreen I Kit (Roche Diagnostics GmbH, Mannheim, Germany) was used. Two µl of cDNA were used as a template for the PCR reaction and incubated in a total reaction volume of 10 µl, containing 1×SYBR Green I Master mix including Taq DNA polymerase, Taq PCR buffer, a dNTP mixture and 1 mM MgCl2, 10 pmol specific EpCAM or GAPDH primers. Forward EpCAM primer (CTG GTG TGT GAA CAC TGC TGG GG), reverse EpCAM primer (TCT CCT TCT GAA GTG CAG TCC GC), forward GAPDH primer (AAA TTG AGC CCG CAG CCT CCC), and reverse GAPDH primer (CCA GGC GCC CAA TAC GAC CAA AT) were synthesized by MWG-BIOTECH AG (Ebersberg, Germany). The PCR conditions were initially 5 min 95°C, followed by 40 cycles of 10 s 95°C, 10 s 67°C (EpCAM) or 72°C (GAPDH) and 20 s 72°C, respectively.

### Protein Extraction and Western Blotting

Total protein extracts from cell lines were obtained by lysing the cells in cold radioimmunoprecipitation assay (RIPA) buffer (50 mM Tris, 2 mM EDTA, 1% NP-40, 0.1% SDS, and 150 mM NaCl) in the presence of protein inhibitor cocktail set I (Calbiochem, La Jolla, USA). After centrifugation to remove cell debris, protein concentrations of the supernatants were measured using the BCA method. 40 µg protein per lane were resuspended in loading buffer (0.5 M Tris-HCl, pH 6.8, glycerol, 10% SDS, 0.5% bromphenol blue, mercaptoethanol) and then subjected to sodium dodecyl sulfate–polyacrylamide gel electrophoresis (8% gels). Subsequently, proteins were blotted onto a nitrocellulose membrane (Hybond®-ECL®, Amersham Biosciences, Freiburg, Germany) following conventional protocols. Finally, blots were blocked in 4% bovine serum albumin/Tris-buffered saline–0.1% Tween 20 (TBS-T) for 30 min at room temperature. Membranes were incubated with primary antibody MOC31 (1 µg/ml, DakoCytomation, Carpinteria, USA) and anti-beta-Actin (0.5 µg/ml, Abcam, Cambridge, UK) over night at 4°C, washed with TBS-T and incubated with a 1∶200 diluted polyclonal goat-anti-mouse antibody (DakoCytomation, Carpinteria, USA) conjugated with horseradish peroxidase for 60 min at room temperature. The bound immune complexes were visualised using ECL Western Blotting Substrate (Pierce, Rockford, USA) and the ChemiDoc XRS System (Bio-Rad, Munich, Germany).

### Flow Cytometry

Cultured cells were detached with Cell Dissociation Buffer (GIBCO™, Carlsbad, US) and incubated on ice for 30 min with MOC31 (DakoCytomation, Carpinteria, USA) primary antibody at a concentration of 1 µg/ml. The corresponding murine isotype control was IgG1 (DakoCytomation, Carpinteria, USA). The cells were washed and the primary antibody was detected with allophycocyanin-conjugated goat-anti-mouse antibody (Becton Dickinson Biosciences, Heidelberg, Germany). Flow cytometry was performed using a FACS CALIBUR flow cytometer (Becton Dickinson, Heidelberg, Germany). Data were analysed using Win MDI 2.9 software.

### Immunocytochemistry

For immunocytochemistry, cells cultured in eight-well chamber slides (Becton Dickinson, Heidelberg, Germany) were washed with PBS, fixed in ice-cold acetone for 2 min, air dried, and blocked with 10% rabbit sera in blocking reagent (TBS). Slides were incubated with MOC31 or IgG1 control antibody, respectively at a dilution of 0.6 µg/ml over night (4°C) then followed by a biotinylated rabbit anti mouse antibody (DakoCytomation) at a dilution of 1∶200 for 30 min. After careful washes in TBS, an incubation with an avidin-alkaline phosphatase complex (ABC kit, Vectastain, Vector, Burlingame, CA) for 30 min followed and thereafter, additional washes in TBS were performed. Alkaline phosphatase activity was visualised using Naphthol-AS-bisphosphate as a substrate and New Fuchsin was used for simultaneous coupling. Slides were counterstained with Mayer’s hemalum diluted 1∶1 in distilled water for ten seconds, blued under running tap water and mounted with Aquatex® (Merck KGaA, Darmstadt, Germany).

### Immunohistochemistry of Tumour Cell Lines Xenotransplanted in SCID Mice

Sections of all primary tumours were drawn from our in house tumour bank. They all had been fixed with 4% formalin and embedded in paraffin wax according to standard procedures. For immunohistochemistry, 5 µm thick sections were cut, dewaxed and treated with bacterial Type XXIV Proteinase (Sigma-Aldrich, Steinheim, Germany) at a dilution of 40 mg/100 ml for 10 min at 37°C. After washing, non-specific binding was blocked by incubating sections in 10% normal rabbit serum (DAKO, Hamburg, Germany) for 30 min at room temperature. The following steps were performed as described above. The intensity (plus signs) and extent (percentage) of the positive areas of five histological sections were determined by visual inspection of three independent observers. The plus sign indicates the intensity of the staining. It ranges from “−” to “+++”. The percentage indicates how many cells were labelled.

### Subcutaneous Implantation of HT29 Cells

To investigate EpCAM binding sites *in vivo*, specific pathogen-free BALB/c SCID (scid/scid) mice were used. The mice were 8–16 weeks old and weighed 20–30 g at the beginning of our experiments. They were housed in filter top cages and provided with sterile water and food ad libitum. For injection, HT29 carcinoma cells were harvested by trypsination and viable cells (5×10^6^) were suspended in 1 ml of cell culture medium. An aliquot of 200 µl of this suspension was injected subcutaneously between the scapulae of each SCID mouse. Six mice bearing carcinomas were included in the experiment when the tumour had reached maximal growth or started to ulcerate.

### Iodination of Antibodies

Iodination of antibodies was done by Iodobeads (Pierce, Rockford, USA). Specifically, 10 µl sodium [125I]Iodine (3.7 MBq/µl, Amersham, UK) was added to 1 mg MOC31 or control IgG1 in 1 ml PBS in the presence of one Iodobead and the reaction was left for 15 min. Free [125I]Iodine was removed by exclusion chromatography through a PD-10 column (GE Healthcare, Buckinghamshire, UK). The protein concentration was determined with the BCA method and the specific activity of the [125I]-EpCAM and [125I]-IgG1 per µg protein was measured by gamma counting (Perkin Elmer, Waltham, USA).

### Application of Radioactive Antibodies

Ten µg of [125I]-labelled antibodies were injected via tail vein into HT29 carcinoma bearing mice. Three mice received control [125I]-IgG1 and three mice the specific anti EpCAM [125I]-MOC31 antibody. After 24 hours blood samples were taken by heart puncture. Afterwards mice were sacrificed, intracardially perfused with NaCl and tumour and organs were removed and weighted. Radioactivity of organs, tumours and blood was determined with the gamma counter. Statistical analyses (Two-way ANOVA) were performed using GraphPad Prism 5 software (GraphPad software, USA).

### Application of Evans Blue

Evans Blue was injected i.v. at a dose of 25 µl (50 mg/ml PBS) per HT29 s. c. bearing mouse 4 hours before sacrifice. Anaesthetised mice were intracardially perfused with 1% BSA in NaCl to remove free dye and afterwards fixed with 4% PFA. Tumours were removed and embedded in 5% agarose gel, cut in 200 µm thick slices with a vibratome (VT1000E, Zeiss, Wetzlar, Germany) and scanned with 2400 dpi on a scanner (Epson, Long Beach, USA).

### Examination of Tumour Blood Vessels

Blood vessels of HT29 tumours were visualized as previously described [25]. Briefly, anaesthetised mice were intracardially perfused consecutively with 2 ml PBS to remove blood, 10 ml of DiI solution with a concentration of 1.2 mg DiI in a mix of PBS and 5% glucose at a ratio of 1∶4, and finally with 5 ml 4% PFA. Tumours and livers were removed and sliced with a vibratome as described above. Laser-scanning confocal microscopy was conducted using a Nikon A1R confocal microscope with a 561 nm diode pumped solid state laser (DPSSL) and with plan apo ×10/0.45 numerical aperture (NA) or plan fluo oil ×40/1.3 NA objective lenses. Image analysis and in particular 3D reconstruction of blood vessels was achieved using NIS-Elements AR software (Nikon Corporation, Japan).

### Measurement of Interstitial Fluid Pressure (IFP)

IFP was measured with a modified wick-in-needle (WIN) technique [26], [27]. In brief, a 21 gauge needle was prepared with a 2±1 mm long side-hole 2 mm distant from the tip. The needle was filled with 4 (6-0 Ethilon) nylon surgical sutures (Ethicon, Norderstedt, Germany) and the tip was closed with acrylic glue (Uhu, Bühl, Germany). The needle was connected to a MSD 100 pressure transducer (SiKA, Kaufungen, Germany) connected by polyethylene tubing filled with sterile, heparinised (10 units/ml) saline. The pressure transducer was connected to handheld double-pressure-meter (model MH3156; SiKA, Kaufungen, Germany) and the signal was recorded with a computer with integrated software (GSOFT3050; SiKA, Kaufungen, Germany). The needle was inserted into the middle of the tumour after the pressure level was set to zero. After reaching a constant pressure level (measured value) the fluid communication between pressure sensor and needle was tested as the tubing was compressed with a screw clamp (arrow b) which results in a sharp increase of the pressure, then an exponential decrease, followed by another period of stabilization before decompressing the tubing (arrow c) which produces a rapid decrease and an exponential increase.

### Magnetic Resonance Imaging

Magnetic resonance imaging examinations were performed using a 7-Tesla Bruker Clinscan animal MRI scanner and a four-channel phased-array surface coil (Bruker BioSpin MRI GmbH, Ettlingen, Germany). All of the mice were anaesthetised continuously with inhaled isofluorane (1.5–2%). During MR examination an integrated thermocouple/heater system to regulate body temperature of mice was used for signal reception. MR imaging was performed using a two-dimensional turbo spin-echo (TSE) sequence (TR/TE/flip = 1480 ms/36 ms/180°, NEX = 16, FOV = 30 mm×26.3 mm) and 24 contiguous 400-µm-thick axial slices were acquired (pixel size = 117×117×400 µm^3^). Total scan time per animal was approximately 8 min. Image analysis was performed using Image Processing and Analysis in Java (ImageJ, version 1.44 p).

### Ethics Statement

The methodology for carrying out the experiment was consistent with the UKCCCR guidelines for the welfare of animals in cancer research. The experiment was supervised by the institutional animal welfare officer and approved by the local licensing authority (Behörde für Soziales, Familie, Gesundheit und Verbraucherschutz; Amt für Gesundheit und Verbraucherschutz, Hamburg, Germany, project no. 92/09).

## Results

### EpCAM Expression of Tumour Cell Lines *in vitro* and *in vivo*

The EpCAM mRNA and protein expression levels of twelve human cancer cell lines representing six different tumour entities were investigated by qPCR, FACS analysis and immunocytochemistry. Furthermore, EpCAM abundance was analyzed by immunostaining of paraffin sections from tumours that were derived from the same cell lines.

**Figure 1:**
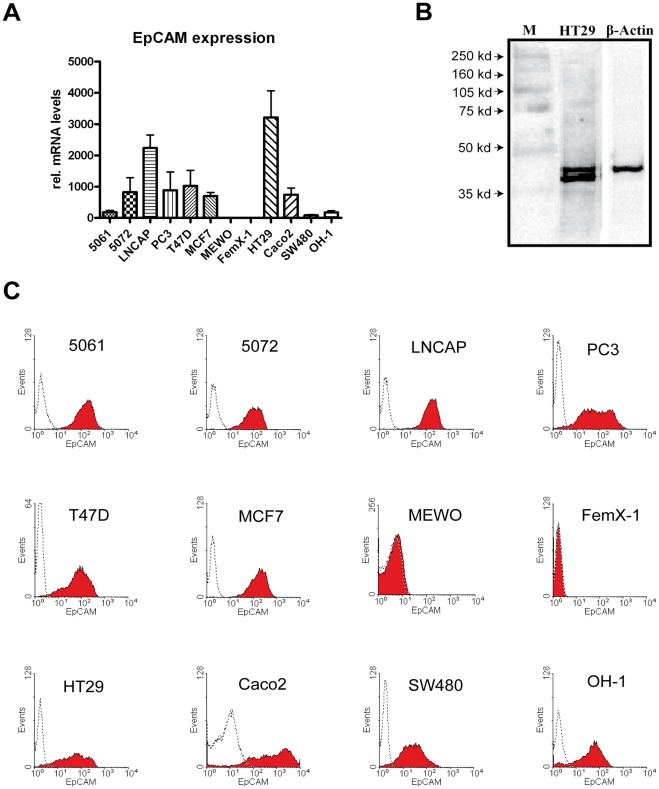
EpCAM expression of human malignant cells. (A) The mRNA of the malignant cell lines 5061, 5072 (pancreatic cancer), LNCAP, PC3 (prostate cancer), FemX-1, MEWO (melanoma), T47D, MCF7 (breast cancer), HT29 (colon cancer) and OH-1 (small cell lung cancer) were relatively quantified by qPCR, using GAPDH for normalization. 5072, LNCAP, PC3, T47D, MCF7, Caco2 and HT29 showed high expression levels of EpCAM mRNA. (B) EpCAM could be detected by Western blot analysis of HT29 cell lysate with a specific binding to antibody MOC31. Beta-actin was used as loading control. (C) EpCAM could positively be detected by flow cytometry analysis with MOC31 on all cancer cell lines, except of FemX-1 and MEWO. Isotype controls are shown as dotted lines.

**Figure 2:**
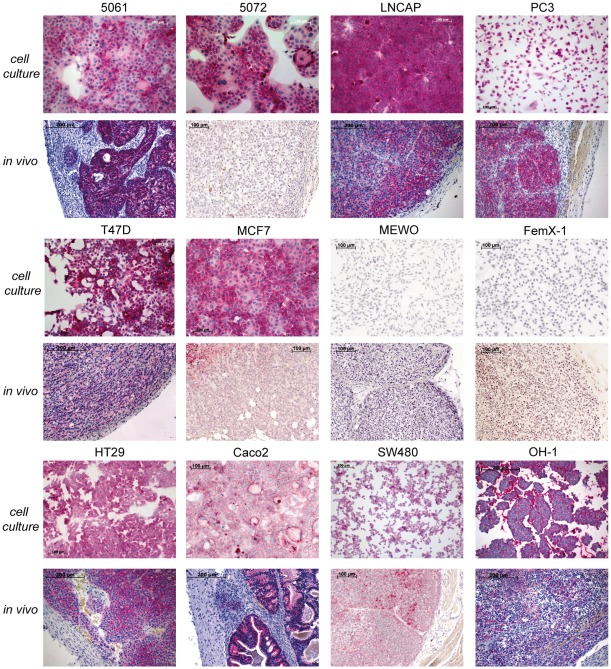
EpCAM protein expression pattern *in vivo* and *in vitro*. 5061, LNCAP, PC3, T47D, HT29, and Caco2 cells showed *in vitro* and *in vivo* strong binding of MOC31 by all cells. In contrast, MCF7, OH-1, SW480 and particularly 5072 cells showed strong MOC31 *in vitro* binding, but little or no binding *in vivo*. MEWO and FemX-1 cells showed no MOC31 binding in vitro and in vivo. (red = MOC31 binding).

The three different colon cancer cell lines displayed high (HT29) to medium (Caco2) to low (SW480) EpCAM mRNA levels *in vitro* as determined by qPCR (see Figure 1A). Analysis of EpCAM protein abundance was performed using the monoclonal MOC31 antibody. The antibody was highly specific and recognized a band of expected size in Western Blot analysis of cell lysate from HT29 cells (see Figure 1B). In addition, we detected a previously described second band presumably representing a glycosylated form of EpCAM [28]. Analysis of EpCAM protein expression at the cell surface by flow cytometry revealed high EpCAM expression level in HT29, Caco2 and to a lesser extent in SW480 cells (see Figure 1C). Comparable immunoreactivity for EpCAM was observed by immunostaining of HT29, Caco2 and SW480 cells *in vitro* and in corresponding tumours *in vivo* (see Figure 2).

The 5061 and 5072 pancreatic cancer cell lines showed medium to low EpCAM mRNA levels *in vitro* (see Figure 1A). Flow cytometry analysis revealed high EpCAM protein expression on the cell surface in both cell lines (see Figure 1C). Similarly, high EpCAM immunoreactivity was also observed in both cell lines when grown *in vitro* (see Figure 2). However, the immunoreactivity of the cells *in vivo* grown as xenografts showed different results: 5061 primary tumours were highly positive for MOC31 binding whereas in 5072 tumours the MOC31 immunoreactivity could not be detected (see Figure 2).

Prostate cancer cell lines displayed *in vitro* medium (PC3) and high (LNCAP) levels of mRNA for EpCAM (see Figure 1A). Cells of both cell lines showed high abundance of membrane bound EpCAM protein and strong MOC31 immunoreactivity of *in vitro* grown cells (see Figures 1C and 2, respectively). Tumours of both cell lines were strongly positive for MOC31 immunoreactivity (see Figure 2).

T47D and MCF7 cell lines derived from breast cancer showed medium levels of EpCAM mRNA expression (see Figure 1A). Both cell lines were strongly positive for EpCAM immunoreactivity in flow cytometry analysis (see Figure 1C) and also after staining of cells *in vitro* (see Figure 2). In contrast, primary T47D and MCF7 tumours grown *in vivo* showed a decreased immunoreactivity for MOC31 binding (see Figure 2).

OH-1 cells representing small cell lung cancer showed *in vitro* low EpCAM mRNA levels (see Figure 1A), although they were highly positive for membrane bound EpCAM protein in flow cytometry analysis (see Figure 1C). These results were confirmed in immunocytochemistry of cells grown under cell culture conditions (see Figure 2). In contrast, OH-1 cells grown *in vivo* in immunodeficient mice displayed a low MOC31 immunoreactivity (see Figure 2).

Our analysis of MEWO and FemX-1 cell lines that are both derived from melanoma revealed that no EpCAM mRNA expression could be detected. Also EpCAM protein immunoreactivity was below detection limits in the melanoma cell lines *in vitro* and *in vivo* (see Figures 1A, 1C, 2).

### EpCAM Binding Detection *in vivo*

**Figure 3:**
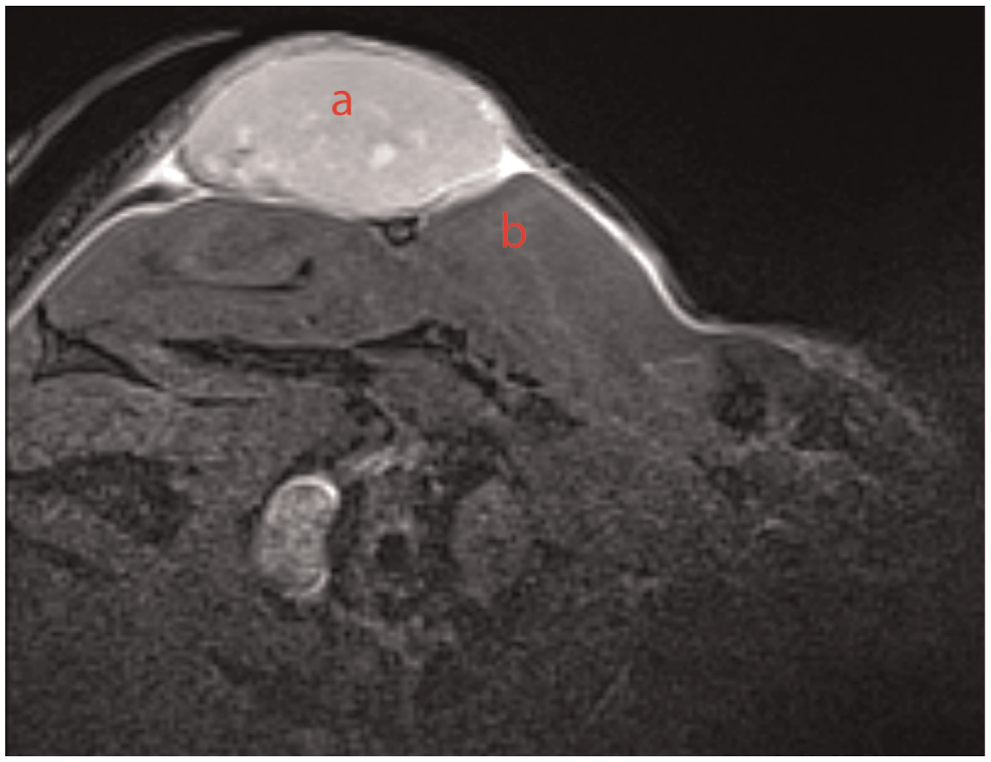
MRI of HT29 xenograft. HT29 colon carcinoma (a) grown at the subcutaneous injection site above muscles (b) appear hyper-intense in two-dimensional turbo spin-echo (TSE) sequence (MR images in axial orientation).

**Figure 4:**
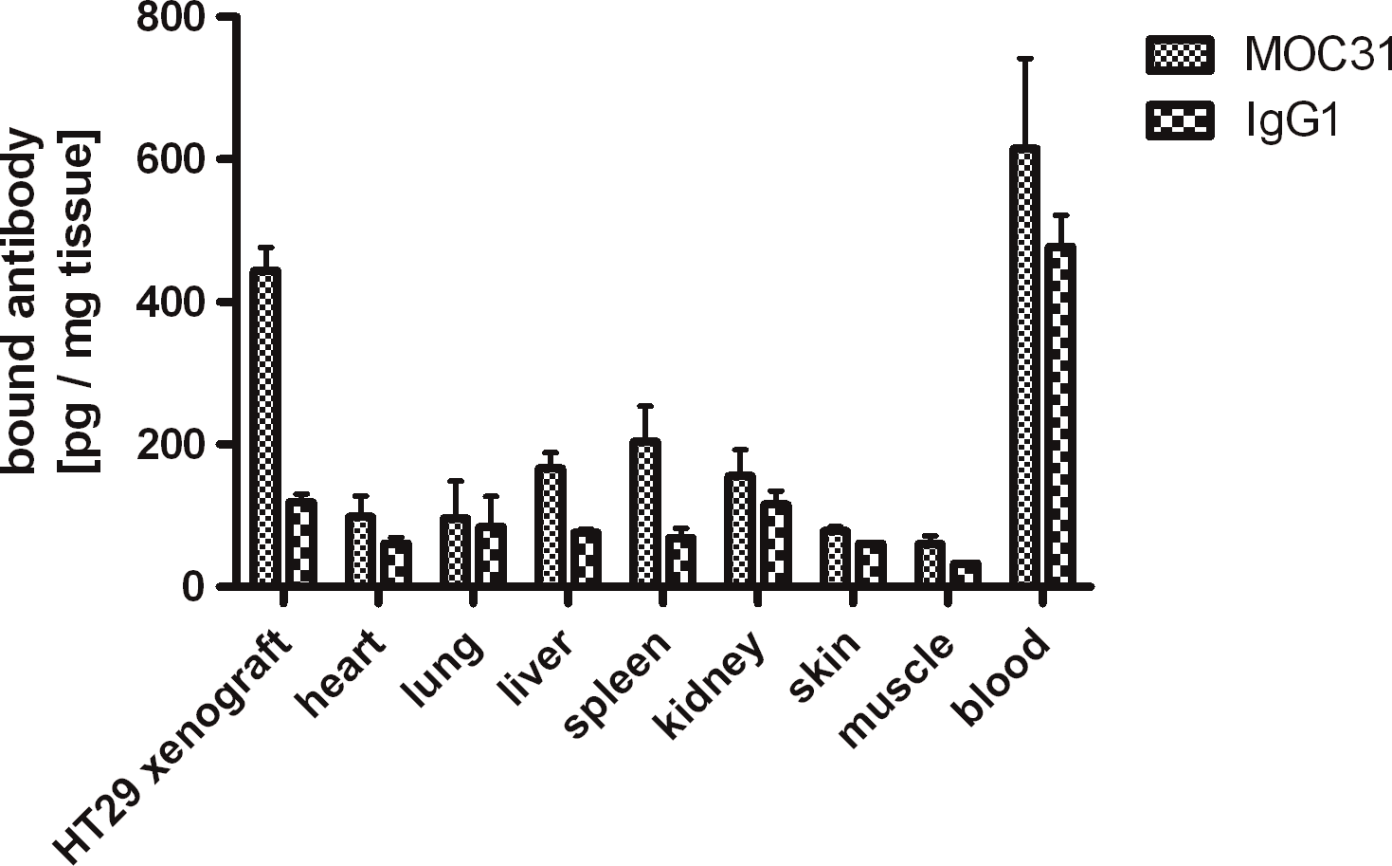
EpCAM *in vivo* binding. [125I]-Labelled specific anti EpCAM MOC31 and non-specific [125I]-labelled IgG1 antibody were used for EpCAM *in vivo* binding in HT29 carcinoma bearing SCID mice. There is a significant difference between specific and control antibody by HT29 carcinoma, but except for spleen and blood not in other organs (two-way ANOVA, P<0.001, n = 3). Standard deviations are indicated by bars.

To analyse whether the EpCAM binding sites detected in tissue sections were also accessible *in vivo*, [125I]-labelled MOC31 mAb and non-specific [125I]-labelled control IgG were injected into the tail vein of SCID mice bearing HT29 colon carcinoma (see Figure 3). A three-fold higher enrichment of the [125I]-MOC31 mAb in the HT29 primary colon carcinoma was detected compared to the [125I]-IgG1 control (see Figure 4). This enrichment was statistically highly significant (two-way ANOVA, P<0.001). Also in spleen a significant three-fold enrichment of the specific [125I]-MOC31 mAb compared with control [125I]-IgG could be observed (two-way ANOVA, P<0.01, see Figure 4).

**Figure 5:**
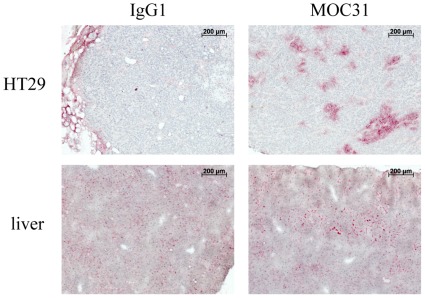
EpCAM *in vivo* detection of HT29 tumour. MOC31-antibody binds to HT29 tumour cells *in vivo* after i.v. injection of 10 µg MOC31mAb in tumour bearing mice, as visualized with subsequent immunostaining against MOC31-antibody (red = MOC31 positive cells) in cryostat sections of the primary tumour. Controls using 10 µg IgG1 mAb confirmed the high specificity of MOC31 binding to tumour cells *in vivo*. The specific antibody MOC31 binding to the tumour cells was restricted to areas of the tumour, which were well supplied with blood vessels. Corresponding liver sections showed the intake of both antibodies by cells of the RES system.

In a parallel experiment the distribution of the injected mAbs 24 hours after application was analysed in histological sections (see Figure 5). No detectable levels of antibody could be found in control animals injected with control IgG1 mAb. In contrast, MOC31 binding could be detected in the vital zones of the HT29 tumour around blood vessels both in the margin of the tumour and in blood vessels located in more central areas of the tumour. However, most parts of the tumour tissue remained unstained.

**Figure 6:**
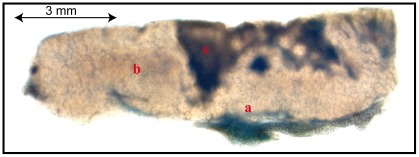
Evans Blue-Albumin complex distribution in vibratome sections of HT29 carcinoma. Evans Blue-Albumin complex positive areas (blue) are recognizable at blood vessels in vital tumour tissue (b), to a higher degree at well perfused areas at the margin of the tumour (a) and in the transition between vital tumour tissue and necrosis (c).

To study the mechanism of the limited MOC31 penetration into the HT29 tumour tissue we investigated the permeability of the tumour blood vessels with an alternative technique. We injected the albumin-binding dye Evans Blue i.v. into mice bearing HT29 colon carcinoma and investigated the dye distribution in vibratome sections of the tumour. Evans Blue-Albumin complexes were present primarily at the margin and around blood vessels in vital zones of the tumour (see Figure 6). Thus, the pattern of Evans Blue-Albumin distribution was similar to the pattern of MOC31 distribution after systemic application of both molecules.

**Figure 7:**
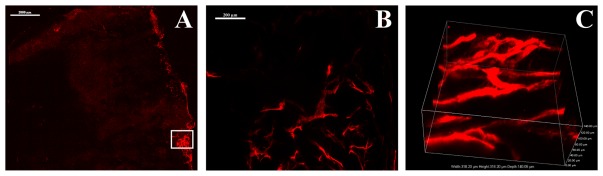
Vasculature of HT29 xenografted tumours visualized by DiI labelling. (A) Unequal distribution of the blood vasculature (red) of a whole HT29 tumour vibratome slice (stitched of ×10 objective lens images). (B) Magnification of the boxed area in A, showing immature blood vessels (projection of a z series with z step size of 2.85 µm over 215 µm with ×10 objective lens). (C) Three-dimensional view of a section of B (z series with z step size of 1.4 µm over 140 µm with ×40 objective lens) showing the irregular structure of blood vessels. All tissues were viewed by confocal fluorescence microscopy (Nikon A1R confocal microscope with plan apo ×10/0.45 numerical aperture (NA) or plan fluo oil ×40/1.3 NA objective lens and with the laser excitation wavelength of 561 nm and the emission of 595). Scale bars: 2000 µm for A, 200 µm for B.

To visualize the distribution and fine structure of HT29 xenograft blood vessels we used an already described DiI perfusion protocol [25]. Well perfused blood vessels were mainly located at the margins and to a lesser extent in the centre of the tumour (see Figure 7A). Higher magnifications and 3D reconstruction of the blood vessels showed that they are highly irregular and could be described as immature (see Figures 7B, C).

**Figure 8:**
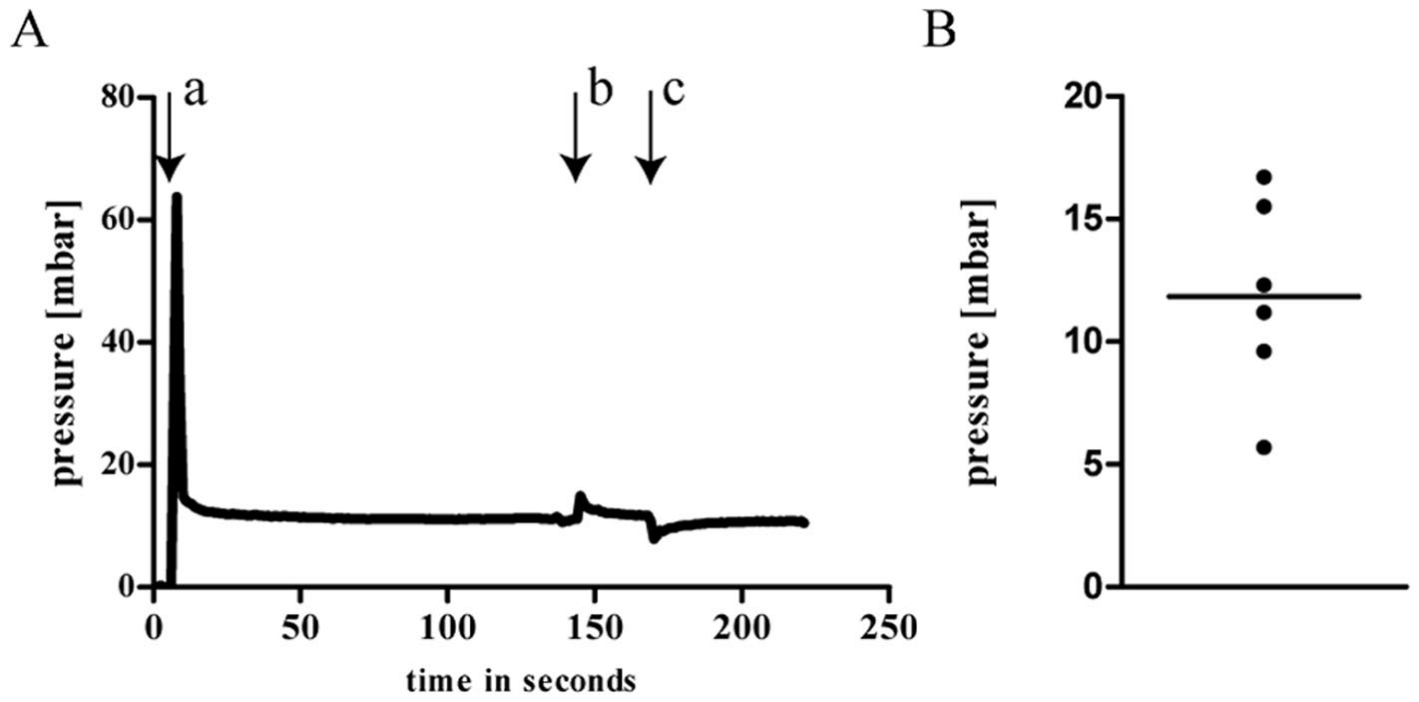
IFP measurements of HT29 xenograft tumours. (A) Typical pressure recording of a HT29 tumour. After adjusting the pressure at 0 mbar and insertion of the needle (arrow a) in the tumour the pressure increased and decreased rapidly and reaches a stable value of 16 mbar. To test the fluid communication between pressure sensor and needle the tubing is compressed with a screw clamp (arrow b) which results in a sharp increase of the pressure, then an exponential decrease, followed by another period of stabilization before decompressing the tubing (arrow c) which produces a rapid decrease and an exponential increase. (B) Measurement of 6 different HT29 tumours showed a mean IFP of 11.9 (+/−4) mbar.

In order to measure the interstitial fluid pressure (IFP) within primary tumours we have established the “wick-in-needle” technique according to the protocol of Boucher et al. In our hands, the interstitial pressure reacted to controlled compression and decompression of the tubing connected to the needle in the expected fashion (see Figure 8A) [27]. We have determined the IFP of six different HT29 tumour xenografts and observed interstitial pressure levels ranging from 6 to 17 mbar with a mean IFP of 11.9+/−4 mbar (see Figure 8B).

## Discussion

This study investigates the possibilities of using anti EpCAM antibodies as a tool for molecular imaging purposes. We used monoclonal antibody MOC31, which was already described by de Jonge in 1993 as a suitable antibody for molecular imaging [29]. Despite of early reports, no clinical development followed with this antibody similar to many other antibodies developed during this time. Therefore, we examined EpCAM expression in a panel of human cancer cell lines and their respective primary tumour xenografts in immunodeficient SCID mice in order to identify a suitable model for the analysis of the accessibility of MOC31 to EpCAM located on the cell surface of cells in primary tumour xenografts. The results of our expression analysis are summarized in Table 1.

**Table 1:** Summary of *in vitro* and *in vivo* EpCAM expression of all cell lines by different methods.

	cell culture			xenografted tumor
	qPCR	FACS positive	immunocytochemistry	immunohistochemistry
5061	+	99%	+++ (95%)	++/+++ (95%)
5072	++	98%	+++ (95%)	− (0%)
LNCAP	+++	99%	+++ (100%)	++/+++ (100%)
PC3	++	96%	++/+++ (100%)	++/+++ (100%)
T47D	++	91%	++/+++ (100%)	+ (100%)
MCF7	++	100%	++/+++ (100%)	−/+ (10%)
MEWO	−	0%	− (0%)	− (0%)
FemX-1	−	0%	− (0%)	− (0%)
HT29	+++	94%	++/+++ (100%)	++/+++ (100%)
Caco2	++	85%	++/+++ (100%)	++/+++ (100%)
SW480	+	94%	++ (100%)	++ (100%)
OH-1	+	93%	++ (100%)	+ (100%)

Note that the highest EpCAM expression both *in vitro* and *in vivo* was observed in the cell lines 5061, LNCAP, PC3, HT29, and Caco2. The intensity (plus signs) and extent (percent) of the positive areas of 5 histological sections were determined by visual inspection of 3 independent observers.

The mRNA expression levels for EpCAM of twelve human cancer cell lines were analyzed *in vitro* by qPCR. The protein abundance on the cell surface was analyzed by flow cytometry and immunocytochemistry under cell culture conditions (see Figure 1 and Table 1). Remarkably, almost all cell lines (except of melanoma cell lines) of the different entities had similar levels of EpCAM protein, although they exhibited different mRNA expression levels. A discrepancy between mRNA levels and protein abundance has already been described for other proteins like Period2 and CEACAM [24], [30]. Differences in mRNA stability are and/or protein turnover are most likely responsible for this phenomenon. However, differences regarding EpCAM mRNA stability among the tumour cell lines used in this study have not yet been investigated. Since the mRNA concentration was not directly related to the measured protein, we were interested in how the protein expression is altered under *in vivo* conditions. For most of the cell lines analyzed we found that levels of EpCAM abundance were similar between cells grown in culture (*in vitro*) and cells grown as a primary tumour in a xenograft model (*in vivo*) judged by immunoreactivity of the MOC31 antibody (see Figure 2 and Table 1). An exception is the pancreatic cell line 5072 in which almost no EpCAM protein expression could be detected *in vivo.* A similar difference in expression levels has already been described for this cell line regarding the abundance of the CEACAM protein family members [24]. However, the fact that the majority of cancers expressed EpCAM *in vivo* and our results indicate that EpCAM expression in tumours is not a limiting factor for its usage for specific targeting.

Next we investigated the binding of the MOC31 antibody to EpCAM expressed on the surface of tumour cells *in vivo*. Injection of radio-labelled MOC31 antibody or non-specific IgG1 antibody, respectively, into HT29 primary colon cancer xenografted SCID mice revealed, that considerable more radioactivity was present if the MOC31 antibody was applied instead of the control IgG1 antibody (see Figure 4). In addition to the enrichment of MOC31 in the tumour itself a significant MOC31 accumulation was observed in the spleen (see Figure 4). This off-target phenomenon was already observed in a study of carcinoma xenografts in nude rats with injected [111In]-labelled MOC31 [29] and occurs in mice as well. The enrichment of the detached [111In]-label in phagocytic cells or Fc-receptor mediated binding of the injected antibody was proposed to be responsible for this effect which has to be taken into account in future studies.

To analyse the MOC31 distribution pattern after i.v. application, we tested the tumour penetration of the MOC31 antibody by immunohistochemistry. Only few regions of the tumour tissues around the blood vessels were positive for injected MOC31 antibody if detected by immunohistochemistry (see Figure 5), whereas the bulk of the tumour tissue was not penetrated by the injected antibody explaining why the tumour enrichment of MOC31 was only about threefold. The same distribution pattern around the blood vessels was observed in another model system using anti CEACAM antibodies in a melanoma xenograft model [24] indicating that this observation of limited antibody distribution is not particular to the HT29 xenograft model. Tumour penetration was additionally investigated with a second neutral marker using an alternative approach. Albumin-bound Evans Blue was visualized on tumour vibratome slices and again could only be detected in the near vicinity of blood vessels (see Figure 6). The pattern of staining related to diffusion and the pattern of distribution of bound MOC31 antibodies to cells within the tumours was similar indicating, that limited diffusion of the antibodies could be the reason for the restricted pattern of targeted EpCAM labelling in MOC31 perfusion experiments. Based on the fact that only a small part of the tumour mass was accessible to the MOC31 antibodies we argue that the actual enrichment of MOC31 in perfused areas of the primary tumours is much higher than estimated since the entire tumour mass was used for the calculation of specific enrichment in our experiments.

To examine why the specific antibody could not reach all the possible binding sites within the tumour we visualized the existing blood vessels of HT29 tumour xenografts. Dense vascularisation was only present at the margins of the tumours. The existing blood vessels were irregular and were denoted as immature (see Figure 7). Immature blood vessels together with sparsely existing lymph vessels were proposed to lead to elevated interstitial fluid pressure (IFP) of the tumours [31]. We therefore measured the IFP in HT29 tumours. Indeed, we could ascertain an increased IFP ranging from 6 to 17 mbar compared to the IFP of normal tissue (see Figure 8). The reliability of IFP measurements could generally be affected by the positioning of the needle within the tumour. In fact, our data show different IFP for six individual tumours analyzed. However, IFP in all tumours was increased compared to control levels that are already described [32]. Thus, in addition to the fact that the vascularisation of the tumours analyzed was inhomogeneous, the increased IFP could contribute to the limited diffusion of antibodies beyond the perivascular area and into the poorly vascularised regions of the tumours [19].

The penetration of antibodies into the tumours could generally also be affected by the tumour microenvironment including the relative perfusion rates as well as the microvessel density and leakiness that were not investigated in this study. However, the hypothesis that limited penetration of molecules into the tumour is mechanistically related to high IFP is supported by data from Tong et al. [31]. Using similar xenograft tumour mouse models they could show that application of a monoclonal antibody directed against VEGF decreased the IFP of the tumours and increased the penetration of tagged BSA used as repoter molecule for tissue penetration. Furthermore, Klosowska-Wardega et al. [33] could show that pharmacological inhibition of PDGF- and VEGF-mediated signaling lead to a reduction of tumour IFP and to an improved therapeutic effect of taxol that needs to penetrate into the tumour in order to exert its effects.

In summary, our results show that antibody penetration into tumours is restricted to areas around blood vessels. This limitation of the penetration correlates with an increased IFP within tumours and severely limits the access of the antibody to the target. Therefore, lowering this IFP could greatly enhance the success of diagnostics and therapeutic interventions based on antibodies [34].
